# Application of Anticonvulsants, Antiepileptic Drugs, and Vitamin C in the Treatment and Analysis of Batten Disease

**DOI:** 10.7759/cureus.21745

**Published:** 2022-01-30

**Authors:** Shreya Reddy, Hetal Brahmbhatt

**Affiliations:** 1 Biology, Creighton University, Omaha, USA; 2 Psychiatry, Mercy General Hospital, Sacramento, USA

**Keywords:** seizures, vitamin c, antiepileptic drug, anticonvulsant, batten disease

## Abstract

Batten disease is a rare group of neurological diseases, specifically called neuronal ceroid lipofuscinosis. This is a genetic disorder and usually manifests during childhood. Batten disease is fatal and there is currently no proven cure. However, there are certain treatment methods that show potential in mitigating the aftermath of the disease. This review will explore the application and effectiveness of antiepileptic drugs, anticonvulsants, and vitamin C in multiple scenarios to treat Batten disease. Anticonvulsants are a broad group of medications that are used to treat epileptic seizures. Epileptic seizures are a big indicator of Batten disease, making anticonvulsants a potential treatment for Batten disease patients. Antiepileptic drugs also work to stop seizures by decreasing neurological excitation, thus for the same reason are often grouped alongside anticonvulsants and are being investigated as a promising way to help Batten disease patients. Vitamin C helps maintain the integrity of several intracellular processes in the central nervous system, which makes it a possible candidate for treating Batten disease. The known effects of anticonvulsants, antiepileptic drugs, and vitamin C on Batten disease are very limited and should be considered more often by healthcare professionals because of their potential effects on patients with Batten disease.

## Introduction and background

Batten disease is a broad cohort of genetic disorders of the nervous system, also known as neuronal ceroid lipofuscinosis (NCL) or ceroid lipofuscinosis, neuronal (CNL). These diseases are fatal and rare. Each specific disease in the group shares a specific defected gene (the gene depends on the specific disease), which interferes with the cell’s ability to recycle certain compounds and molecules [1]. Most commonly, Batten disease refers to the juvenile onset of CNL. Common symptoms of Batten disease are seizures, vision loss, abnormal movements of the body, and insomnia [1]. Over time, the symptoms can progress into worsened seizures and loss of language and motor skills. Eventually, Batten disease patients become blind, bedridden, and lose all cognitive function [2]. The potential risk of death depends on the exact form of Batten disease and the age of onset of Batten disease [3]. Of every 100,000 babies born in the United States (US), two to four have inherited Batten disease from their biological parents [4]. Both males and females have an equal chance of inheriting Batten disease [4-5]. 

There is currently no cure for any of the forms of Batten’s disease. However, in 2017, the FDA approved an enzyme replacement therapy for ceroid lipofuscinosis, neuronal type 2 (CLN2) disease, in which patients present a tripeptidyl-peptidase 1 deficiency [5]. However, the enzyme replacement therapy procedure approved by the FDA is only for the CLN2 form of Batten disease and is not applicable to the other diseases that are encompassed in the group. Additionally, the enzyme replacement therapy procedure costs $702,000 yearly, meaning it costs $27,000 per biweekly infusion [6]. There are other treatment methods used to alleviate the symptoms of Batten disease, which are less expensive and more accessible. This review will examine anticonvulsants, antiepileptic drugs, and vitamin C and their effects on Batten disease patients. 

This research paper aims to explore the effects of anticonvulsants, antiepileptic drugs, and vitamin C on the symptoms of Batten disease. A literature search of articles published between 1988 and 2021 using PubMed was performed using the keywords “Batten disease”, “anticonvulsant”, “antiepileptic drug”, “vitamin C”, and “seizures”. Cases that included infants, the elderly, allergies related to anticonvulsants, antiepileptic drugs, and vitamin C, existing history of other previous neural surgeries, and head trauma were excluded. Titles and abstracts were analyzed to ensure patients were not in a compromised state prior to treatment of Batten disease. Only articles that were available online and in English were included in this review. The articles reviewed included case studies and group studies. The articles were analyzed for the type of treatment used for Batten disease, the dosage of the treatment method, and the overall results on the patient. Results from the studies done were analyzed in a mixed-methodology manner, using both quantitative and qualitative analysis to create a holistic review. The quantitative analysis included percentages of effectiveness in some of the treatments, such as how many patients had improvements with elements of their condition, such as seizures. The qualitative analysis included descriptions of visual elements of Batten disease such as the frequency and severity of seizures. A total of 56 articles were identified of which 40 were actually reviewed, due to the aforementioned criteria.

## Review

Anticonvulsants

Anticonvulsants are medications used to counteract seizures [7]. Anticonvulsants work by calming brain hyperactivity [8]. Anticonvulsant drugs decrease signals in the brain that increase the probability of seizures and also help reduce the frequency and severity of seizures, should they occur. They also prevent seizures from spreading within the brain through the blockage of sodium channels. The nerves can be damaged by a wide variety of factors, such as injury or exposure to neurotoxins. Anticonvulsants interfere with the overactive transmission of pain signals sent from damaged nerves, easing the patient’s pain. The general role of anticonvulsants is to act as both a mood stabilizer and to treat the patient’s neuropathic pain. Many antiepileptic drugs block sodium channels or enhance γ-aminobutyric acid functions, which has anti-seizure and anti-anxiety effects by blocking neurotransmissions [8]. Through the blockage of sodium or calcium channels, there is a significant reduction in the release of excitatory molecule glutamate, whose release is considered to be elevated in epileptic activities. A common anticonvulsant is lamotrigine, which is used to treat bipolar disorder and certain types of seizure disorders [8]. Valproate is another type of anticonvulsant that can also be helpful in mitigating migraine pain, in addition to treating bipolar disorder and seizures [8]. 

Antiepileptic drugs

Antiepileptic drugs are another potential treatment method for Batten disease. Antiepileptic drugs also work to stop seizures and are often grouped together with anticonvulsants. Antiepileptic drugs affect electrical activity in the neurons by changing ion concentrations in the cell membrane [9]. They are also known for altering chemical transmission between neurons by affecting neurotransmitters in the synapse. These medications are said to help control seizures in seven out of 10 people [9]. They are usually prescribed to children with recurrent seizures, making them a probable treatment for patients with Batten disease. Isradipine is an antiepileptic drug that has neuroprotective effects. Isradipine is able to create some dopamine fibers and cell bodies at concentrations achievable in humans, suggesting that isradipine is a potentially viable neuroprotective agent that can help patients with neurological disorders, such as Batten disease [9].

Vitamin C

Vitamin C is also a potential candidate for treatment for Batten disease, as vitamin C is involved in the physiology of the nervous system, including the support and the structure of the neurons [10]. This is a molecule found in many foods and is noted as an essential component of the enzymatic production of some neurotransmitters. vitamin C specifically aids in the processes of differentiation, maturation, and neuronal survival. Vitamin C is also considered to be a critical antioxidant in the brain [11]. Many neurological disorders, including Batten disease, are known for excessive creation of free radicals, and the highest concentration of vitamin C is typically found in the brain and neuroendocrine tissues. Therefore, it can be suggested that Vitamin C could be a factor in changing the course of neurological issues and be of therapeutic use to patients [12]. Vitamin C deficiency has been linked to nerve dysfunction [13]. Additionally, intracellular vitamin C catalyzes several functions in the central nervous system, such as antioxidant protection, peptide amidation, myelin formation, synaptic potentiation, and protection against glutamate toxicity [14]. 

Dosage of anticonvulsants, antiepileptic drugs, and vitamin C

Many anticonvulsant treatment plans involve introducing the anticonvulsant to the patient slowly to minimize potential side effects. For instance, it takes a couple of months to increase the adult dosage to 200 milligrams per day. The typical adult dosage tends to be 0.5-1 milligram three times per day [15]. Children are usually treated based on their weight and height. The dosage is incredibly important because if they are not carefully planned out, side effects can be devastating for the patient. An overdose of anticonvulsants can lead to serious issues such as central nervous system depression, ataxia, and nystagmus. In the studies encompassed in this review, many patients who were taking lamotrigine started off with smaller dosages such as 0.1-0.5 milligrams per kilogram of body weight per day [16-17]. The dosage of antiepileptic drugs also varies between adults and children. The usual adult dosage of antiepileptic drugs is 500-1000 milligrams twice a day. Many patients typically start off around 250 milligrams twice a day for a week, then increase by increments of 250 milligrams per week until the desired results are achieved. The dosage of antiepileptic drugs is also critical as it can heavily affect the patient. If too many antiepileptic drugs are ingested, serious complications such as respiratory depression, apnoea, coma, and ventricular arrhythmias can occur [18]. Vitamin C dosage is also important to the overall health of the patient. The typical dosage of vitamin C is 5-75 milligrams for children, 75 milligrams for adult women, 90 milligrams for adult men, and 85-120 milligrams for women who are pregnant or breastfeeding [19-21]. Although an excessive amount of vitamin C is unlikely to be very harmful, overdose side effects include nausea, heartburn, insomnia, and abdominal pain [22-23]. One study in this review administered vitamin C at 14.3 milligrams/kilogram of body weight per day in conjunction with sodium selenite [24]. Another set of experiments treated patients with 0.008-0.025 milligrams/kilogram of body weight per day [25-26]. One case study included in this review treated patients with vitamin C that was naturally made by the bodies of the patients with the recorded concentration: 103.6-148.7 micromoles per liter [27]. 

Results of anticonvulsants

The results of the treatment with anticonvulsants show some potential. In a study with 16 patients, they were treated with lamotrigine at a dosage of 0.5 milligram/kilogram of body weight for 15 patients, and one patient had their dose increased over two weeks to 4.7 milligrams/kilogram of body weight [16]. Lamotrigine proved to help nearly all patients’ seizures for two years [16]. Additionally, in another experiment in this review, lamotrigine was administered to 28 patients starting with dosages of 0.1-0.5 milligrams/kilogram of body weight per day, then increased every two weeks up to 1.25-15 milligrams/kilogram of body weight per day. The results showed that lamotrigine helped 23 out of the 28 patients [17]. With a different setting, 60 patients were treated with anticonvulsants valproate, lamotrigine, phenobarbital, and felbamate Valproate was administered at 10 milligrams/kilogram of body weight per day, lamotrigine at 0.5 milligrams/kilogram of body weight per day, phenobarbital at 1 milligram/kilogram of body weight per day, and felbamate at 5-10 milligrams/kilogram of body weight per day [28]. With the usage of different anticonvulsants, there was an overall 50% decrease in seizures. Clonazepam is another anticonvulsant that has been experimented with [29-30]. Zonisamide is an anticonvulsant that was given to patients in a study at 100-600 milligrams daily for two to three years and showed promise at effectively treating epilepsies [31-32]. One study combined anticonvulsants levetiracetam (1000 milligrams twice a day), sodium valproate (400 milligrams twice a day), lamotrigine (125 milligrams twice a day), zonisamide (100 milligrams in the morning, 150 milligrams at night), and diazepam (10 milligrams, given when required). For the next three months, the patient did not experience major seizure activity of sporadic jerks in the palms [33]. Another example of combining anticonvulsants is one study that used barbiturates and valproate. The results showed that barbiturates caused erythrocyte ethanolamine phosphoglycerides and phosphatidylethanolamine plasmalogens to decrease, but the valproate treatment had no effect on erythrocyte ethanolamine phosphoglycerides and phosphatidylethanolamine plasmalogen levels [34]. In a group experiment, 35 patients were treated with valproate given at 60-1000 milligrams daily. Valproate had a variety of effects, the most common being: valproate-induced hyperammonemic encephalopathy, drowsiness, and decreased seizures [35].

In a different situation, valproate was again tested with patients, being administered at 63-75 milligrams/liter. In the patient who received the lower dose, there were only mild and intermittent effects on the Batten disease onset [36]. In the other patient, there was only a mild reduction of symptoms [36]. Anticonvulsants have also been shown to cause increased fluidity in membranes when membrane fluidity in juvenile Batten disease patients tends to be decreased [37]. Valproic acid (VPA) is a derivative of valproate that was also experimented with as an anticonvulsant. In one assay, patients were treated with VPA at 15 milligrams/kilograms of body weight per day. Shortly after the first couple of treatments, there was a report of breakthrough seizure activity especially in the mornings, indicating treatment was unsuccessful [38]. While there are multiple assays indicating that anticonvulsants serve as a treatment method for Batten disease successfully, there are others showing that anticonvulsants can fail at being effective [39-40]. For instance, phenytoin is an anticonvulsant that was tested, and the results show that phenytoin doesn’t cause a statistical difference in the activity of free radical metabolism of patients with Batten disease [41]. However, since the majority of experiments indicated some level of success, it can be deduced that anticonvulsants could very well be a viable solution to Batten disease. 

Results of antiepileptic drugs and vitamin C

There is promising, yet limited (due to the criteria mentioned for studies encompassed in this piece of literature), evidence of the success of antiepileptic drugs in treating Batten disease as shown by Table 1. One study combined the antiepileptic drugs amiodarone, cis diltiazem HCl, felodipine, flavoxate HCl, and isradipine. The effects from the treatment of multiple antiepileptic drugs simultaneously show significant lowering in calcium levels in ceroid lipofuscinosis, neuronal type 3 (CLN3) small interfering ribonucleic acid (siRNA) knockdown cells, which reduces defects in the CLN3 cells, leading to less presence of Batten disease [42]. Vitamin C also shows possible capability in treating Batten disease. In one case, 74 patients were given vitamin C at 0.008-0.025 milligrams/kilogram of body weight per day. The effects on female patients were significant, as the treatment decreased menstruation periods in women, which correlated with a decrease in seizures [25]. Another assay with vitamin C given at 0.008-0.025 milligrams/kilogram of body weight per day resulted in either no progress or only slight progress observed in mental/neurological symptoms of Batten disease [26]. In one scenario, vitamin C that was naturally made by the bodies of the patients was used to observe its effects on neurological problems. The average concentration of vitamin C was 103.6-148.7 micromoles/liter. The results indicated that vitamin C levels are decreased in some patients who have neurological disorders like Batten disease [27]. While results from antiepileptic drugs are limited and vitamin C has variable results, both show promise in becoming a commonplace treatment for Batten disease patients. 

**Table 1:** Summary of previous research of the manipulation of anticonvulsants, antiepileptic drugs, and vitamin C in the comprehension and care of Batten disease N/A: not applicable; HCl: hydrochloric acid; siRNA:  small interfering ribonucleic acid; CLN3: ceroid lipofuscinosis, neuronal type 3

Author (Year) and Sample Size	Treatment Type and Dosage	Results
Aberg et al. (1997), 16 [16]	Anticonvulsant (lamotrigine), 0.5 milligram/kilogram of body weight for 15 patients, one patient had their dose increased over two weeks to 4.7 milligrams/kilogram of body weight	Lamotrigine had a favorable effect in nearly all of the patients over two years
Aberg et al. (1999), 28 [17]	Anticonvulsant (lamotrigine), started at 0.1-0.5 milligram/kilogram of bodyweight per day, then increased every two weeks up to 1.25-15 milligram/kilogram of body weight per day	Lamotrigine had a good effect in 23/28 patients
Clausen et al. (1988), 73 [24]	Anticonvulsant sodium selenite and vitamin C; Sodium selenite: 94.5 microgram/kilogram of body weight per day, Vitamin C: 14.3 milligram/kilogram of body weight per day	Lower selenium levels in patients were found to correlate to neurological issues similar to Batten disease By taking vitamin C and sodium selenite daily, some abnormalities of Batten disease were reduced in all patients
Santavuori et al. (1988), 74 [25]	Vitamin C 0.008-0.025 milligrams/kilogram of body weight per day	Decreased menstruation periods in women, which correlated with a decrease in seizures
Santavuori et al. (2009), 125 [26]	Vitamin C 0.008-0.025 milligrams/kilogram of body weight per day	No progress or only slight progress was observed in mental/neurological symptoms of Batten disease
Sass et al. (1999), 2 [27]	Vitamin C (naturally made by the body of the patients); concentration: 103.6-148.7 micromoles/liter	Vitamin C levels are decreased in some patients who have neurological disorders like Batten disease
Aberg et al. (2000), 60 [28]	Anticonvulsants valproate, lamotrigine, phenobarbital, felbamate; valproate: 10 milligram/kilogram of body weight per day, lamotrigine: 0.5 milligram/kilogram of body weight per day, phenobarbital: 1 milligram/kilogram of body weight per day, felbamate: 5-10 milligram/kilogram of body weight per day	An overall decrease in 50% of the seizures was observed
Boustany (1992), 80 [29]	Anticonvulsants clonazepam and valproic acid; dosages are N/A	N/A
Conry (2002), N/A [31]	Anticonvulsants valproic acid, ethosuximide, lamotrigine, benzodiazepines, piracetam, zonisamide; zonisamide: 100-600 milligrams daily for two-three years	Zonisamide shows promise in treating epilepsies, valproic acid is effective but should be used with caution in young children
Georgiou et al. (2020), 1 [33]	Anticonvulsants levetiracetam (1000 milligrams twice a day), sodium valproate (400 milligrams twice a day), lamotrigine (125 milligrams twice a day), zonisamide (100 milligrams in the morning, 150 milligrams at night), diazepam (10 milligrams, given when required)	For three months after treatment, the patient did not experience major seizure activity of sporadic jerks in the palms
Kohlschütter et al. (1993), 11 [34]	Anticonvulsants barbiturates and valproate; dosages: N/A	Barbiturates caused erythrocyte ethanolamine phosphoglycerides and phosphatidylethanolamine plasmalogens to decrease; Valproate treatment had no effect on erythrocyte ethanolamine phosphoglycerides and phosphatidylethanolamine plasmalogen levels
Larsen and Ostergaard (2014), 35 [35]	Anticonvulsant valproate 60-1000 milligrams daily	Valproate had a variety of effects, the most common being: valproate-induced hyperammonemic encephalopathy, drowsiness, and decreased seizures
Johannsen et al. (2016), 2 [36]	Anticonvulsant valproate at 63-75 milligrams/liter	On the patient who received the lower dose, there were only mild and intermittent effects on the Batten disease onset; On the other patient, there was only a mild reduction of symptoms
Kohlschütter et al. (1988), 17 [37]	Anticonvulsant; specific drug and dosage: N/A	Anticonvulsants caused increased fluidity in the membrane when membrane fluidity in juvenile Batten disease patients tends to be less
Patel (2008), 1 [38]	Anticonvulsant valproic acid 15 milligrams/kilograms of body weight per day	Report of breakthrough seizure activity especially in mornings
Kohlschütter (1988), 29 [39]	Anticonvulsant; specific drug and dosage: N/A	Concluded that anticonvulsants should be used judiciously
Yamaguchi et al. (2020), 35 [40]	Anticonvulsant; specific drug and dosage: N/A	A true relationship between anticonvulsant and Batten disease could not be established
Maertens et al. (1995), 7 [41]	Anticonvulsant phenytoin; dosage: N/A	Phenytoin doesn’t cause a statistical difference in the activity of free radical metabolism of patients of Batten disease
An Haack et al. (2011), N/A [42]	Antiepileptic drugs amiodarone, cis diltiazem HCl, felodipine, flavoxate HCl, isradipine	All show significant lowering in calcium levels in CLN3 siRNA knockdown cells, which reduces defects in the CLN3 cells, leading to less presence of Batten disease

## Conclusions

From all of the data of the experiments incorporated in this piece of literature, it is comprehensible that anticonvulsants can serve as a potential method to expand on our limited knowledge and treatment methods of Batten disease. Anticonvulsants are a valuable treatment method because of their ability, as demonstrated by the majority of the data, to stop epileptic seizures. Furthermore, antiepileptic drugs and vitamin C have also proved useful in the treatment of Batten disease. The concentration of vitamin C in the brain and neuroendocrine tissues have shown to make a difference in patients with Batten disease and is useful overall to the human nervous system, particularly the central nervous system. Antiepileptic drugs are helpful with seizures as they decrease membrane excitability by interacting with neurotransmitter receptors or ion channels. There is still much work to be done, especially with antiepileptic drugs and vitamin C, in doing more trials with different dosages, and obtaining more data with those treatment methods. Anticonvulsants, combined with vitamin C and antiepileptic drugs, could potentially serve as the backbone treatment for Batten disease. Further studies should be conducted with vitamin C, antiepileptic drugs, and anticonvulsants to understand their long-term effects on mitigating the neurological results of Batten disease.
